# Resting Energy Expenditure and Body Composition in Overweight Men and Women Living in a Temperate Climate

**DOI:** 10.3390/jcm9010203

**Published:** 2020-01-11

**Authors:** Marcos Martin-Rincon, Mario Perez-Valera, David Morales-Alamo, Ismael Perez-Suarez, Cecilia Dorado, Juan J. Gonzalez-Henriquez, Julian W. Juan-Habib, Cristian Quintana-Garcia, Victor Galvan-Alvarez, Pablo B. Pedrianes-Martin, Carmen Acosta, David Curtelin, Jose A.L. Calbet, Pedro de Pablos-Velasco

**Affiliations:** 1 Department of Physical Education, University of Las Palmas de Gran Canaria, Campus Universitario de Tafira, s/n, 35017 Las Palmas de Gran Canaria, Canary Islands, Spain; marcos.martinrincon@gmail.com (M.M.-R.); marioperezvalera@gmail.com (M.P.-V.); moralesalamo.d@gmail.com (D.M.-A.); ismaelperezsuarez@gmail.com (I.P.-S.); cdoradogarcia@gmail.com (C.D.); jjhulpgc@gmail.com (J.W.J.-H.); cristiancafd@outlook.com (C.Q.-G.); victor_galvan@hotmail.es (V.G.-A.); 2 Research Institute of Biomedical and Health Sciences (IUIBS), University of Las Palmas de Gran Canaria, Paseo Blas Cabrera Felipe “Físico” (s/n), 35017 Las Palmas de Gran Canaria, Canary Islands, Spain; gonzalezhenriquez@gmail.com (J.J.G.-H.); ppedmar@yahoo.es (P.B.P.-M.); carmen_acosta87@hotmail.com (C.A.); davidcurtelin@gmail.com (D.C.); 3 Department of Mathematics, University of Las Palmas de Gran Canaria, Campus Universitario de Tafira, s/n, 35017 Las Palmas de Gran Canaria, Canary Islands, Spain; 4 Department of Endocrinology and Nutrition, Hospital Universitario de Gran Canaria Doctor Negrín, Calle Plaza Barranco de la Ballena, s/n, 35010 Las Palmas de Gran Canaria, Canary Islands, Spain; 5 Department of Physical Performance, The Norwegian School of Sport Sciences, Postboks, 4014 Ulleval Stadion, 0806 Oslo, Norway

**Keywords:** overweight, obesity, exercise, resting energy expenditure

## Abstract

This study aimed to determine whether the measured resting energy expenditure (REE) in overweight and obese patients living in a temperate climate is lower than the predicted REE; and to ascertain which equation should be used in patients living in a temperate climate. REE (indirect calorimetry) and body composition (DXA) were measured in 174 patients (88 men and 86 women; 20–68 years old) with overweight or obesity (BMI 27–45 kg m^−2^). All volunteers were residents in Gran Canaria (monthly temperatures: 18–24 °C). REE was lower than predicted by most equations in our population. Age and BMI were similar in both sexes. In the whole population, the equations of Mifflin, Henry and Rees, Livingston and Owen, had similar levels of accuracy (non-significant bias of 0.7%, 1.1%, 0.6%, and −2.2%, respectively). The best equation to predict resting energy expenditure in overweight and moderately obese men and women living in a temperate climate all year round is the Mifflin equation. In men, the equations by Henry and Rees, Livingston, and by Owen had predictive accuracies comparable to that of Mifflin. The body composition-based equation of Johnston was slightly more accurate than Mifflin’s in men. In women, none of the body composition-based equations outperformed Mifflin’s.

## 1. Introduction

The assessment of daily energy expenditure is essential in clinical nutrition to elaborate diets and nutritional treatments according to the patients’ needs. Resting energy expenditure (REE) represents 60–80% of the daily energy expenditure in most patients [1], and should be determined for an accurate dietetic prescription. In general, REE is measured by indirect calorimetry (IC), which requires sophisticated equipment and time-consuming procedures. Alternatively, REE may be predicted from anthropometric and/or body composition data using prediction equations [2,3]. The use of prediction equations entails associated errors due to the population-specificity of the equations and the procedures used in their development and validation [2,4,5]. Some of the most used equations in the clinical setting, as the Harris and Benedict 1984 [6] and FAO/WHO/UNU [7], have been shown to overestimate REE in overweight and obese subjects, in contrast to the Mifflin et al. equation [8] which is more accurate in subjects with overweight or obesity [9,10,11].

Several factors may influence REE [12], including sex [13], race [14], age [15], body composition [16], sleep duration [14], energy balance [16], food composition [17], physical activity [18], circadian rhythms [19], and obesity-related comorbidities [11,20,21,22]. For these reasons, some equations specific to overweight and obese patients have been proposed. To avoid the potential influence of comorbidities in REE-predictive equations, Lazzer et al. [23] developed specific equations for obese subjects after excluding the patients with overt metabolic and/or endocrine diseases (e.g., diabetes, hypothyroidism, hypertension) and those who were taking regular medications known to influence REE. Since the main determinant of REE is fat-free mass (FFM) [16,22,24,25], several equations have been proposed to account for differences in FFM [8,22,23,26,27,28,29,30,31,32,33]. Nevertheless, REE equations based on body composition data do not seem to outperform those based exclusively on anthropometric variables [23,30]. 

Despite often being overlooked, environmental conditions may also influence REE [5,34]. For example, it has been reported that the body mass index (BMI) is more elevated in humans residing in higher environmental temperatures [35]. Henry and Rees, using published REE data obtained in tropical populations observed that the WHO/FAO/UNU equation overestimates REE by 8–11.5% in people living in tropical regions [5]. Nevertheless, the Henry and Rees’s equation was developed using data from different studies, obtained mostly from normal-weight people of varied ethnicities, and it is uncertain whether this equation can be applied to an overweight and moderately obese Caucasian population [5]. Moreover, the Henry and Rees’s equation has not been validated. 

Gran Canaria is located in a subtropical region with mean monthly temperatures between 18 and 24 °C in the coldest and warmest months, respectively. The prevalence of overweight (40.0%) and obesity (20.1%) in Canary Islands [36,37] is high compared to other European regions and countries [38]. Although environmental conditions may be counteracted in part by behavioral factors (amount of clothing, air-conditioning, reduced physical activity, etc.), it has been suggested that increased time spent in the thermal comfort zone may reduce REE contributing to the high prevalence of obesity in subtropical regions [34,39]. If this was the case, overweight and obese people living in Gran Canaria should have an REE falling below that predicted by most equations. In fact, it remains unknown which is the best REE-predictive equation to use in overweight and obese adults living in a temperate weather all year round. 

Therefore, the aim of this investigation was double. Firstly, to determine whether the measured REE in overweight and obese patients living in a temperate climate all year round falls below the predicted values given by most equations, particularly by equations specifically developed for overweight and obese patients. Secondly, to ascertain which is the most accurate equation in overweight and obese participants living in a temperate climate. 

## 2. Material and Methods

### 2.1. Patients

One hundred and seventy-four participants with overweight or obesity volunteered to participate in a clinical trial aiming to reduce body weight with exercise and a low-calorie diet (ISRCTN11049554). As inclusion criteria, men and women living permanently in Gran Canaria had to be aged 18 to 70 years with a BMI ≥27 kg m^−2^, without medical contraindications to exercise and smoking less than six cigarettes per day. Patients with glucose intolerance or type 2 diabetes (if diagnosed within the last five years) were also admitted. More details on inclusion/exclusion criteria can be found in ISRCTN registry. Data were collected from June to October 2016. The study was conducted in accordance with the Declaration of Helsinki and received the approval of the Local Ethical Committee (Ref. 140187). All subjects received oral and written information about the purposes, risks and benefits of the study before they provided their written consent.

### 2.2. General Procedures

For this purpose, subjects reported to the laboratory between 7:00 and 9:30 A.M., following a 12-h overnight fast. Smokers were asked to refrain from smoking for at least 8 h before the tests. All subjects were requested not to exercise and to refrain from drinking alcohol and caffeinated drinks during the 48 h preceding the tests. Upon arrival, their body weight and height were measured to the nearest 0.1 kg and 0.1 cm, respectively. Measurements were performed while subjects wore light clothes and no shoes using a balance scale (Seca, Hamburg, Germany) calibrated using certified calibration masses of class M1. After that, their body composition was determined by dual-energy X-rays absorptiometry (Lunar iDXA, General Electric, Madison, WI, USA), as previously reported [40]. Then, their blood pressure was measured (Omron M3 Intellisense HEM-7131-E, Hoofddorp, Netherlands) after a 5-min seated-period following the recommendations of the American Heart Association [41]. This was followed by the assessment of their resting metabolic rate (RMR) by indirect calorimetry, as explained in the next section. 

### 2.3. Resting Metabolic Rate

Resting metabolic rate (RMR) was measured by indirect calorimetry with a facemask (Vmax N29; SensorMedics, Yorba Linda, California, USA or Vyntus CPX; Jaeger-CareFusion, Hoechberg, Germany) at an ambient temperature of 23–26 °C [42]. The metabolic carts were calibrated immediately before each test according to the manufacturer’s instructions, using certified high-grade calibration gases. The Vmax N29 SensorMedics has been validated for indirect calorimetry by the ethanol-burning test [30]. In our laboratory, both metabolic carts slightly overestimated the stoichiometric RQ of butane combustion, the Vmax N29 by 2.8% and the Vyntus by 1.5%, with a coefficient of variation below 1% in both cases [43]. For further analysis, all Vyntus CPX data were transformed into Vmax N29 data, using values obtained with both analyzers in a parallel cross-calibration study. 

To assess the RMR, subjects were placed in a supine position on a comfortable laboratory stretcher provided with a pillow for 30 min. Participants were instructed to lie motionless, avoid talking and remain awake during the measurement, which was carried out in a well-ventilated room while maintaining a quiet environment. Oxygen uptake (VO_2_) and carbon dioxide production (VCO_2_) were measured breath-by-breath for 20 min after an initial 10-min habituation period using a facemask. For further analysis, the data were averaged every 20 s. All 20-s averages with VO_2_ values deviating from the mean more than two SD were discarded. Then, the mean VO_2_ and VCO_2_ values recorded during a 10-min period with steady VO_2_ were averaged to calculate the daily resting energy expenditure [44]. The respiratory quotients during RMR assessment ranged from 0.7–0.99, i.e., within the physiological range.

### 2.4. Physical Activity

Participants were equipped with a Garmin Vivofit activity tracker (micro-electromechanical triaxial accelerometer) (Garmin International Inc., Olathe, KS, USA) to record their physical activity during at least four consecutive days, including two weekend days. Participants’ characteristics (gender, age, weight and height) were added to the Garmin Connect website and synchronized with the activity tracker as recommended by the manufacturer. Subjects were instructed to wear the device on the non-dominant wrist and set up the sleep-tracking mode. 

### 2.5. Statistical Analysis

The statistical analyses were performed using IBM SPSS v.21.0 for Apple Computers (IBM, New York, NY, USA). The values reported are means ± standard deviations. The measured REE with indirect calorimetry was compared with the REE calculated with the predictive equations (Table S1) using a paired two-tailed *t*-test. A two-tailed unpaired *t*-test was used to compare men and women. 

The agreement between measured and estimated REE values from the predictive equations was assessed by determining the bias in absolute values and as a percentage of the measured value and the corresponding limit of agreement (upper limit of agreement (ULA) = bias + 1.96 × SD; lower limit of agreement (LLA) = bias − 1.96 × SD). We also determined the concordance correlation coefficient (CCC) as a measure of agreement [45]. The percentage of subjects whose predicted REE value fell within ± 10% of the measured REE was taken as a measure of accuracy [2]. All analyses were performed for the whole group and separated by sex. The root mean squared deviation (RMSD) was calculated, which is the root of the squared difference between predicted and measured REE, with low values suggesting good agreement. Although RMSD assesses the absolute error in each measure, it does not take into account that women have a lower body size, while the mean absolute percentage error (i.e., (|measured-predicted REE |*100/measured REE)) (MAPE) does. The absolute percentage errors were compared between sexes with a student *t*-test. The same approach was used to compare anthropometric with body composition-based equations using a paired *t*-test, separately in men and women. Stepwise multiple regression analysis was used to determine the best predictors of REE in our population. Two models were tested: model 1 included weight, height, age, BMI, and sex; and model 2 (based on body composition variables), age, sex, FFM and fat mass, according to the variables included in published equations (see Table S1). Statistical significance was set at *p* < 0.05 for all tests. 

## 3. Results

The descriptive characteristics of the study population are reported in Table 1 and Table 2. The male and female group had similar age and BMI, while women had a greater percentage of body fat than men. Daily physical activity was similar in both sexes. Predicted REE by equations based on anthropometric and body composition variables (Table S1) was compared with measured REE. 

The results obtained with equations based on anthropometric variables combined or not with age and sex are reported for the overall study population (Table 3), as well as for men (Table 4) and women (Table 5) separately. The results obtained with REE equations based on body composition data are reported in Table 6.

**Figure 1:**
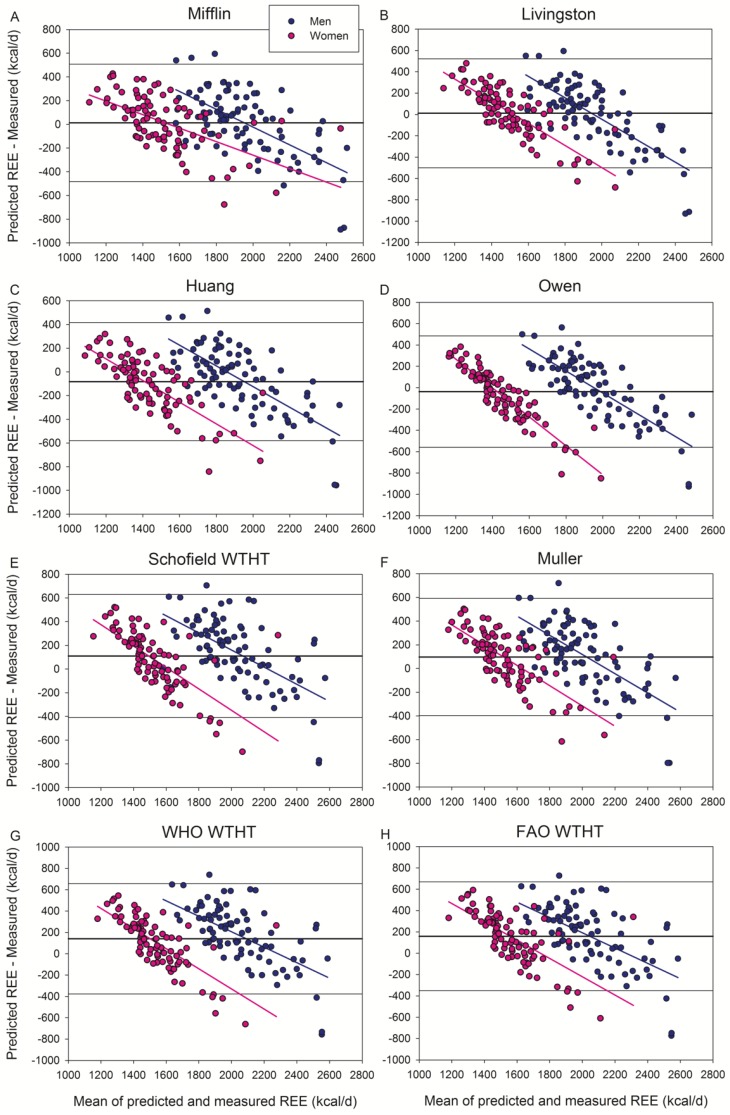
Bland–Altman plots displaying the agreement between measured and predicted REE by the equations of (**A**) Mifflin, (**B**) Livingston, (**C**) Huang, (**D**) Owen, (**E**) Schofield WTHT, (**F**) Muller, (**G**) WHO WTHT, and (**H**) FAO WTHT. All these equations included obese subjects in their populations. The thick continuous line indicates the mean value of the differences between predicted and measured REE (bias). The thin lines delimit the 95% confidence interval. All the regression lines were statistically significant at *p* < 0.001, indicating a systematic bias. Note that “Y” and “X” axes have different scales.

**Figure 2:**
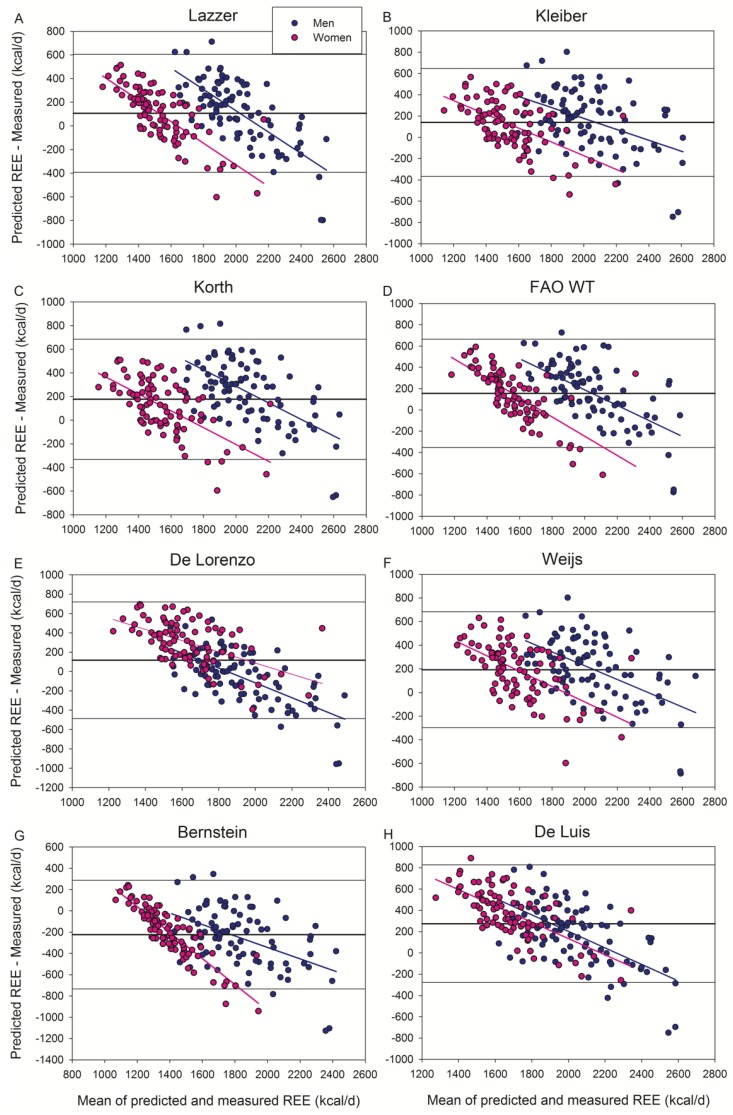
Bland–Altman plots displaying the agreement between measured and predicted REE by the equations of (**A**) Lazzer, (**B**) Kleiber, (**C**) Korth, (**D**) FAO WT, (**E**) De Lorenzo, (**F**) Weijs, (**G**) Bernstein, and (**H**) De Luis. All these equations included obese subjects in their populations. The thick continuous line indicates the mean value of the differences between predicted and measured REE (bias). The thin lines delimit the 95% confidence interval. All the regression lines were statistically significant at *p* < 0.001, indicating a systematic bias. Note that “Y” and “X” axes have different scales. De Luis equation was obtained using a portable, hand-held device (MedGem) less accurate than metabolic carts.

**Figure 3:**
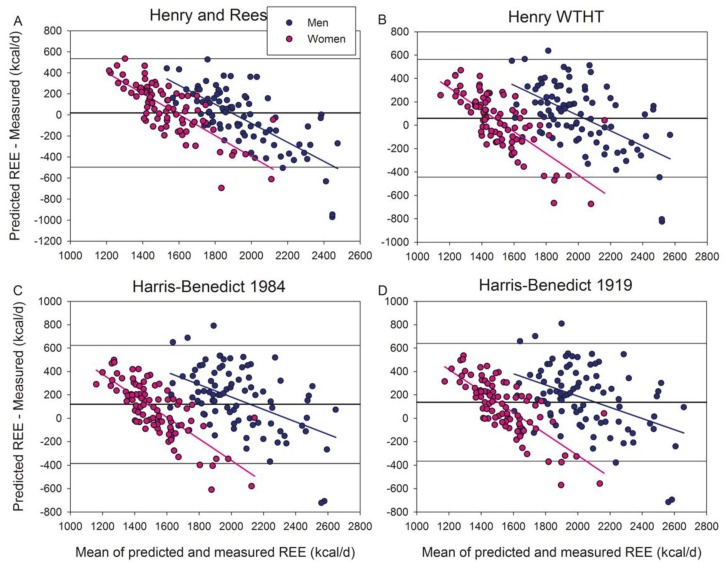
Bland–Altman plots displaying the agreement between measured and predicted REE by the equations of (**A**) Henry and Rees (tropical populations), (**B**) Henry WTHT, (**C**) Harris–Benedict 1984, and (**D**) Harris–Benedict 1919. All these equations did not include obese subjects in their populations. The thick continuous line indicates the mean value of the differences between predicted and measured REE (bias). The thin lines delimit the 95% confidence interval. All the regression lines were statistically significant at *p* < 0.001, indicating a systematic bias. Note that “Y” and “X” axes have different scales.

Bland and Altman’s plots are presented in Figure 1, Figure 2 and Figure 3, as well as in Figures S1–S3. When all subjects were analyzed conjointly, the equations of Mifflin et al. and Livingston et al. gave the best scores of accurate predictions (51% of patients) with a non-significant bias of 0.6–0.7% (Figure 1A,B).

Of the seventeen additional equations tested here, the Huang et al. (Figure 1C) and Bernstein et al. (Figure 2G) equations underestimated (4.8% and 13.0%, respectively), while the other equations overestimated (3.4–15.9%) REE. The equations that did not include obese subjects overestimated REE (Figure 3), except the Henry and Rees equation developed for tropical inhabitants which had a non-significant bias (+1.1%) (Figure 3A). 

In men (Table 3), the equation of Henry and Rees (developed for tropical populations) was accurate (non-significant bias of −1.1%) and was the equation having the highest proportion of accurate predictions (53.5%). The equations of Livingston, Owen and Mifflin were also accurate (with non-statistically significant biases) and able to accurately predict REE in at least 50% of subjects. Non-significant differences were observed in mean absolute percentage errors between the equations of Henry and Rees, Livingston, Owen, and Mifflin in men. However, the CCC was slightly worse for the Henry and Rees equation than for the Mifflin et al. equation. The Mifflin et al. equation had a non-significant 0.5% bias, being accurate in 50% of men. The rest of the equations were less accurate with a predominance of equations overestimating the measured REE (14/2, equations over/underestimating). 

The equations of Henry and Rees, De Lorenzo, De Luis and Lazzer (body composition) 2010 were more accurate in men than women, while the rest of the equations were similarly accurate in both sexes (Table 4 and Table 5). The Mifflin equation gave the most accurate REE predictions in women, with a non-significant 1.1% bias and more than 52% accurate predictions. The Mifflin equation was followed by Livingston and Henry WTHT equation, which also had a small (1.3–2.2%) non-significant bias and scored 48.8% accurate predictions. The Henry and Rees equation for tropical populations overestimated REE in women by 4.7%. The other equations had a bias above 5%, with a predominance of equations overestimating the measured REE (14/3, equations over/underestimating). The FAO WT, Weijs, De Lorenzo, and De Luis equations overestimated REE in women by more than 10%.

**Figure 4:**
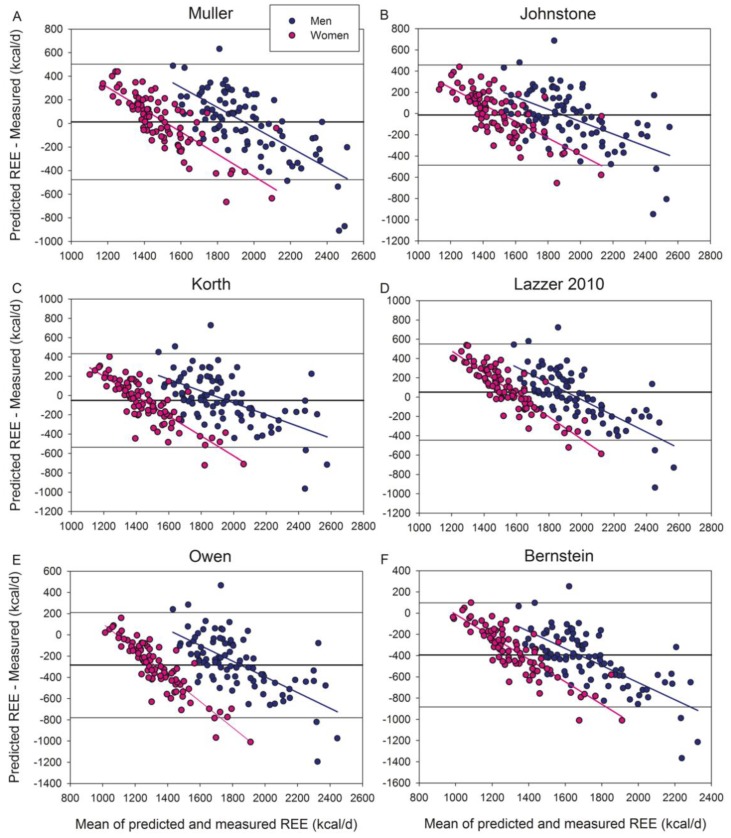
Bland–Altman plots displaying the agreement between measured and predicted REE by the body composition-based equations of (**A**) Muller, (**B**) Johnstone, (**C**) Korth, (**D**) Lazzer 2010, (**E**) Owen, and (**F**) Bernstein. All these equations included obese subjects in their populations. The thick continuous line indicates the mean value of the differences between predicted and measured REE (bias). The thin lines delimit the 95% confidence interval. All the regression lines were statistically significant at *p* < 0.001, indicating a systematic bias. Note that “Y” and “X” axes have different scales.

Of the equations based on body composition variables (Table 6) (Figure 4 and Figure 4S), Muller, Lazzer 2010, Johnstone, and Korth equations scored accurate predictions in at least 55% of men, while only Muller’s reached 50% accurate predictions in women. The body composition-based equations of Muller, Korth, Owen, and Bernstein had a lower MAPE than their respective anthropometry-based equations, both in men and women. In men, Lazzer 2010 body composition equation had a lower MAPE than its anthropometry-based equation (Table 6) and was as accurate as the Mifflin equation in our male population. Of the body composition-based equations, Johnstone’s had a significantly lower MAPE compared with Livingston, Owen, and Mifflin, in men. The MAPEs of Johnstone and Henry and Rees equations were similar. None of the body composition-based equations outperformed the Mifflin equation in women.

Multiple regression analysis showed that REE can be predicted in our population with equation 1 or 2, as follows

REE (kcal/d) = 15.78 × Weight (kg) + 169.07 × sex + 143.87(1)

(R = 0.76, R^2^ = 0.58, SEE = 252.7, *p* < 0.001); age, BMI and height were excluded from the model (sex: 0 = women; 1 = men).

REE (kcal/d) = 24.91 × FFM (kg) + 7.88 × FM (kg) + 87.35(2)

(R = 0.79, R^2^ = 0.62, SEE = 241.4, *p* < 0.001); age and sex excluded from the model (sex: 0 = women; 1 = men).

## 4. Discussion

In agreement with our hypothesis, most equations overpredicted REE in overweight and obese humans living in a temperate climate all year round, including the equations specifically developed for overweight and obese patients. This study also shows that the most accurate equations to predict REE in overweight and obese Caucasian patients living in an all-year-round temperate climate are the Henry and Rees, Johnstone, Mifflin, Owen and Livingston equations in men, and the Mifflin equation in women. Our study also indicates that the Johnstone equation outperforms the rest of the equations based on body composition variables in men. Interestingly, none of the body composition-based equations outperformed the Mifflin equation in women. Out of the most used equations in the clinical setting, i.e., the Harris and Benedict [46], the Owen et al. [31,47], the WHO/FAO/UNU [48], and the Mifflin et al. [2,8], in women, only the Mifflin, and in men, the Henry and Rees, Owen, and the Livingston equations had no significant bias and scored accurate predictions in approximately 50% of subjects in our population. This close-to-50% accuracy is what can be expected from the best predictive equations in overweight and obese patients [30]. Given the extended use of Mifflin’s equation and the fact that none of the anthropometry-based equations were more accurate than the Mifflin equation in men nor women in overweight and moderately obese patients living in a temperate climate, the Mifflin equation emerges as the equation of choice for similar populations. Nevertheless, given the large inter-individual variability in REE, whenever possible, REE must be measured rather than estimated from predictive equations.

Reduced thermogenesis in overweight and obese humans living in a temperate climate may contribute to obesity [49,50]. Cold exposure increases REE in humans by diverse mechanisms, including activation of brown adipose tissue [51], increased muscle thermogenesis [52], and fat cell thermogenesis [53]. Chronic activation of these thermogenic processes by daily cold exposure during several days is required to induce a sustained increase in REE in humans and cold acclimatization [54]. Since in the Canary Islands cold days are exceptional and rarely last for more than two weeks, the predominantly temperate and warm weather may result in a sustained attenuation of REE, being this the most plausible reason why the majority of REE predictive equations overestimate the measured REE in our overweight and obese population. Nevertheless, it should also be taken into account that, in men, several equations, not specifically developed for patients living in tropical regions, were predicting REE with similar or slightly better accuracy than the equations developed including tropical or subtropical populations. The latter would suggest that part of the smaller REE observed in our population may just reflect differences in body composition.

The Livingston et al. [55] equation performed well in our male and female population, although the CCC was slightly below that of the Mifflin et al. equation [8]. The excellent performance of the Livingston and Mifflin equations may be in part due to the use of metabolic carts from SensorMedics in these two studies, as in the present investigation. Moreover, in the case of the Livingston equation, almost half of the subjects used in developing their predictive equation were residing in Dallas area, also a region with temperate/warm weather most of the year. Despite the inherent variability that may affect the equation proposed by Henry and Rees for tropical populations, due to the use of data obtained in different populations, with different habits (diet, physical activity, sleep patterns, etc.) and body composition, this equation achieved a good agreement with our measured REE, particularly in men.

Lazzer et al. developed equations specific for severely obese patients, after excluding the patients with comorbidities that could influence REE [23]. Lazzer 2010 equations were obtained using the same type of metabolic cart (Vmax N29, SensorMedics) as in the present study. Moreover, Canary Island ancestry is somewhat similar to that of the North of Italy, making this equation and population ideal for comparison with our population living in a temperate climate. Nevertheless, Lazzer 2010 anthropometry-based equations overestimated REE in men (+6.0%) and women (+6.3%) when applied to our population. Although some comorbidities and drugs have been associated with increased REE in obese patients [11], our main results were not altered when the subjects on medical treatment were excluded from the analysis. This was somehow expected since both diabetes [22], and hypertension [20,21] have been associated with increased REE and could not explain the decreased REE of our population. Moreover, none of our diabetic patients were on insulin therapy.

Previous studies have reported lower accuracy [8,22,26,27,28], or no clear advantage of REE equations derived using body composition data [23,29,30,31,32,33] when compared with equations solely based on anthropometric measurements. This has been attributed to the variability and diversity of procedures applied to measure FFM and FM (bioelectrical impedance, anthropometry, hydrodensitometry, air-displacement plethysmography, whole-body water assessment with deuterium, total-body potassium, and dual-energy x-ray absorptiometry). Here, DXA was used to determine FFM and FM; a procedure also applied by Johnstone et al. [56] and Korth et al. [57]. This may be one of the reasons why Johnstone’s and Korth’s equations predicted the REE of our men with acceptable accuracy. However, our results also indicate that the equations developed using bioelectrical impedance [23,29] are as accurate as Johnstone’s in our population. In contrast, the body composition-based equations of Owen and Bernstein, both based on hydrodensitometry [31,33,47], underestimated the measured REE markedly. In agreement with Marra et al. [30], our findings indicate that obese-specific equations do not necessarily predict the REE of an obese population more accurately than those obtained from normal-weight subjects.

The fact that our population may have slightly lower REE than others may explain why three of the four equations developed for non-obese subjects were also accurate in our subjects. The reason why the Henry and Rees equation performed worse in our female population might originate from the small number of women used to develop the equation (98 women for the 30–60 age range) and the fact that we studied women with overweight and obesity.

The Harris-Benedict equation, being the oldest in use, has been subjected to more validation studies than any other equation [9]. In general, these validation studies have shown a 5–10% overestimation of REE [8,29,31,47,58,59,60,61,62]. In agreement, we have also observed that the Harris and Benedict equations (1919 and the 1984 modifications) overestimated REE by 5–9% in our overweight/obese population. This overestimation could be even greater in severely obese patients [2].

Our results aligned well with the systematic review by Frankenfield et al. [2] and the recent study by Cancello et al. [11], the latter including 4247 morbidly obese patients. Both studies [2,11] concluded that the best equation to estimate the REE of obese patients is the Mifflin equation, even when comorbidities are present, although Mifflin’s equation may lose accuracy in patients with hypertension [11]. In our population, 44 patients were hypertense, but they were on treatment, which may have counteracted some of the hypertension-related mechanisms increasing REE, for example, increased sympathetic activity [20,21]. In any case, the accuracy of the Mifflin equation was similar in our overweight/obese subjects to that reported for other populations with no or several comorbidities [11].

Even though the Mifflin et al. equation was slightly less accurate in our male population, when using the concordance correlation coefficient as a criterion, the Mifflin et al. equation achieved rather good scores both in men and women, compared to the rest of the equations tested here and had no bias. Moreover, the Mifflin equation had the lowest MAPE in men, and one of the smallest in women. The latter combined with the extended use of the Mifflin et al. equation are strong arguments for defending its use in our population, as well as in similar patients with overweight or moderate obesity (up to BMI close to 40 kg m^−2^) [30,63,64,65], and especially in patients from regions with a temperate climate. Besides, recent reports have shown that the Mifflin equation is robust and may even improve its accuracy in severely obese patients with comorbidities [11].

This study has several limitations. We enrolled a heterogeneous group of overweight and obese patients without excluding subjects due to comorbidities, which in one side may improve the transferability to the general obese population but on the other may have resulted in greater variability and imprecision [11]. This was necessary due to the numerous measurements performed which included assessment of body composition by DXA, aerobic fitness (exercise test), oral glucose tolerance tests, and assessment of daily physical activity. The final sample was similar in size to that of Johnstone et al. [32] who also determined body composition by DXA, and developed an equation that performed very well in our male population. We used two different metabolic carts during the study, but both were verified with a butane combustion test and cross-calibrated, as previously reported [43,66]. The observations made in this investigation are specific to our Caucasian populations and cannot be generalized to other ethnic groups. Despite these limitations, the standard error of the estimate of equation 1 and 2 was similar to that reported for the equations developed for overweight and obese populations [23,26,65].

## 5. Conclusions

In conclusion, the best equation to predict resting energy expenditure in overweight and moderately obese men and women living in a temperate climate all-year-round are the Mifflin et al. equations, while the equations developed by Henry and Rees, Livingston et al. and Owen et al. have predictive accuracies comparable to that of Mifflin et al. equation in men. In men, only the body composition-based equation of Johnston was slightly more accurate than the Mifflin et al. equation and is advisable when trustworthy body composition information is available.

## Figures and Tables

**Table 1:** Characteristics of the study population.

	Men (*n* = 88)					Women (*n* = 86)					*p*-Value
	Mean	±	SD	Range		Mean	±	SD	Range		
				(Min-Max)					(Min-Max)		
Age (years)	40.8	±	9.3	22.0	67.9	42.6	±	10.4	19.8	65.2	0.231
Weight (kg)	105.2	±	13.0	80.8	144.9	86.4	±	10.5	70.8	135.1	<0.001
Height (cm)	177.0	±	6.5	161.1	190.4	162.3	±	5.5	149.9	177.5	<0.001
BMI (kg m^−2^)	33.5	±	3.0	26.9	41.3	32.7	±	2.9	27.7	45.4	0.086
Body fat (%)	37.0	±	4.6	26.3	51.3	46.9	±	3.7	38.9	56.1	<0.001
Total lean mass (kg)	62.8	±	8.0	48.1	89.7	43.2	±	4.4	33.1	56.4	<0.001
Distance (km d^−1^)	7.5	±	3.3	1.4	19.1	6.8	±	2.7	2.0	13.8	0.129
Steps d^−1^	11,160	±	3731	1767	24,044	11,491	±	3455	5345	21,747	0.553

BMI: body mass index; Distance: distance walked or ran every day; Step d^−1^: number of steps performed every day.

**Table 2:** Medical treatments, menopausal state, and smoking.

	Men	Women	Total
Smokers ^a^	10	13	23
Asthma ^b^	3	8	11
Type 2 diabetes ^c^	6	4	10
- Metformin	4	2	6
Hypertension	21	23	44
- Diuretics and ACE inhibitors or ARE blockers	8	21	29
- Betablockers	3	4	7
- Calcium channel blockers	5	5	10
Hypercholesterolemia	24	14	38
- Statins	8	7	15
- Fenofibrate		1	1
Oral contraceptives		9	9
Postmenopausal		20	20

^a^ Less than 6 cigarettes per day; ^b^ on occasional treatment with asthma inhalers; ^c^ Four subjects with type 2 diabetes were controlled with diet and exercise; ACE: Angiotensin converting enzyme; ARE: Angiotensin Receptor.

**Table 3:** Assessment of resting energy prediction equations in overweight and obese adults (88 men and 86 women) from the population of Gran Canaria.

	REE (kcal/d)			*t*-Test	Bias	LLA	ULA	Bias	LLA	ULA	Maximal Error		Prediction			
Equation	Mean	SD	RMSD	*p*-Value	kcal/d	kcal/d	kcal/d	%	%	%	Under %	Over %	CCC	Accurate %	Under %	Over %
REE Measured	1727	389												100		
Mifflin	1737	282	252	0.51	12.8	−483	508	0.7	−27.3	28.8	−37	35	0.72	51.1	20.1	28.7
Livingston	1735	257	261	0.59	10.7	−501	522	0.6	−28.2	29.5	−38	38	0.68	51.1	18.4	30.5
Henry WTHT	1784	307	263	0.003	59.3	−444	563	3.4	−25.0	31.9	−36	37	0.72	50.6	13.8	35.6
Henry and Rees *	1752	228	265	0.35	19.1	−497	535	1.1	−28.0	30.2	−40	41	0.65	48.5	18.9	32.5
Huang	1642	294	267	0.000	−82.1	−581	417	−4.8	−33.0	23.5	−48	30	0.71	47.7	34.5	17.8
Owen	1687	290	268	0.07	−37.3	−560	485	−2.2	−31.8	27.5	−46	32	0.69	47.7	28.2	24.1
Schofield WTHT	1835	310	286	0.000	110.5	−408	629	6.4	−22.7	35.5	−34	41	0.68	45.4	10.3	44.3
Muller	1822	289	270	0.000	97.9	−397	593	5.7	−22.3	33.7	−33	39	0.70	44.8	11.5	43.7
H–B 1984	1844	329	283	0.000	119.4	−386	624	6.9	−21.4	35.2	−32	42	0.70	44.3	9.2	46.6
H–B 1919	1861	324	291	0.000	136.8	−368	641	7.9	−20.4	36.3	−30	43	0.69	44.3	7.5	48.3
Lazzer 2010	1830	281	275	0.000	106.0	−393	605	6.1	−22.1	34.4	−32	40	0.68	44.3	10.9	44.8
WHO WTHT	1864	312	298	0.000	139.8	−377	657	8.1	−20.9	37.2	−32	42	0.67	44.3	7.5	48.3
FAO WTHT	1869	314	301	0.000	144.5	−374	663	8.4	−20.7	37.4	−32	42	0.66	43.7	7.5	48.9
Kleiber	1864	320	294	0.000	139.9	−367	647	8.1	−20.3	36.6	−29	43	0.68	41.4	9.2	49.4
Korth	1901	339	313	0.000	176.9	−332	686	10.3	−18.0	38.6	−32	45	0.67	40.8	6.9	52.3
FAO WT	1880	289	302	0.000	155.5	−354	665	9.0	−19.8	37.9	−30	44	0.64	39.7	8.6	51.7
De Lorenzo	1841	184	328	0.000	117.0	−486	720	6.8	−28.0	41.6	−39	51	0.45	39.7	15.5	44.8
Weijs	1917	301	315	0.000	193.0	−297	683	11.2	−16.8	39.2	−32	47	0.64	37.4	6.3	56.3
Bernstein	1500	263	343	0.000	−223.9	−734	287	−13.0	−41.9	15.9	−50	21	0.56	35.6	58.6	5.7
De Luis	1999	209	393	0.000	274.8	−278	827	15.9	−16.3	48.2	−29	61	0.43	27.0	5.2	67.8

WTHT: Equation including weight and height; H–B: Harris and Benedict equation; WT: equation including weight; REE: resting energy expenditure; SD standard deviation; Bias: mean difference between the measured REE and the predicted value; LLA: lower limit of agreement; ULA: upper limit of agreement; Bias %: mean bias in %; LLA %: lower limit of agreement in percentage; ULA % upper limit of agreement in percentage; CCC, concordance correlation coefficient; Accurate %: percentage of subjects in which the error of the predictive equation was within 10% of the measured value. Under %: percentage of subjects underestimated by the predictive equation with an error > 10% of the measured value; Over %: percentage of subjects overestimated by the predictive equation with an error > 10% of the measured value. * Equation developed for tropical populations (*n* = 86). Equations highlighted did not include obese patients. The background colour is used to identify some of the equations.

**Table 4:** Assessment of resting energy prediction equations in overweight and obese men (*n* = 88) from the population of Gran Canaria.

	REE (kcal/d)			*t*-Test	Bias	LLA	ULA	Bias	LLA	ULA	Maximal Error		Prediction				
Equation	Mean	SD	RMSD	*p*-Value	kcal/d	kcal/d	kcal/d	%	%	%	Under %	Over %	CCC	MAPE	Acc %	Und %	Over %
REE Measured	1958														100		
Henry and Rees *^&^	1930	156	280	0.33	−29.8	−578	518	−1.5	−28.3	25.2	−40	30	0.43	10.5	53.5	24.4	22.1
Livingston	1955	141	283	0.95	2.0	−555	559	0.1	−27.3	27.5	−38	35	0.39	11.3	53.4	19.3	27.3
Henry WTHT	2038	196	283	0.004	85.5	−447	618	4.4	−21.8	30.6	−33	35	0.49	12.1	52.3	11.4	36.4
De Lorenzo ^&^	1894	176	277	0.047	−58.4	−593	476	−3.0	−29.3	23.4	−39	30	0.47	10.5	52.3	28.4	19.3
Huang	1895	158	277	0.050	−57.7	−592	477	−3.0	−29.2	23.3	−39	30	0.45	10.5	51.1	28.4	20.5
Owen	1952	133	275	0.99	−0.3	−543	543	−0.02	−26.7	26.6	−38	32	0.41	11.1	50.0	20.5	29.5
Mifflin	1962	179	273	0.76	9.1	−528	546	0.5	−26.1	27.1	−36	34	0.48	10.1	50.0	22.7	27.3
Muller	2064	169	294	0.000	111.6	−425	648	5.7	−20.7	32.1	−32	39	0.43	12.9	47.7	10.2	42.0
Lazzer 2010	2070	154	298	0.000	117.5	−423	658	6.0	−20.6	32.7	−32	38	0.40	13.2	45.5	11.4	43.2
Schofield WTHT	2096	185	312	0.000	143.0	−404	690	7.3	−19.5	34.2	−31	38	0.41	13.8	45.5	8.0	46.6
H–B 1919	2123	228	324	0.000	170.9	−372	714	8.8	−18.0	35.5	−28	43	0.45	14.5	44.3	4.5	51.1
WHO WTHT	2129	185	329	0.000	176.4	−370	723	9.0	−17.9	35.9	−30	40	0.39	14.9	44.3	4.5	51.1
FAO WT	2116	184	321	0.000	163.7	−381	708	8.4	−18.3	35.1	−30	39	0.40	14.4	44.3	6.8	48.9
H–B 1984	2116	218	318	0.000	163.2	−374	701	8.4	−18.1	34.9	−28	42	0.45	14.2	43.2	5.7	51.1
FAO WTHT	2133	187	331	0.000	180.7	−367	728	9.3	−17.7	36.2	−30	40	0.38	15.1	43.2	4.5	52.3
Kleibler	2113	222	324	0.000	160.9	−393	715	8.2	−19.0	35.5	−29	42	0.44	14.4	43.2	6.8	50.0
Weijs	2149	207	331	0.000	196.7	−329	722	10.1	−15.8	36.0	−26	42	0.43	15.1	42.0	5.7	52.3
De Luis ^&^	2125	180	335	0.000	172.3	−395	740	8.8	−19.3	36.9	−29	46	0.35	15.2	39.8	8.0	52.3
Korth	2189	188	360	0.000	236.4	−299	772	12.1	−14.4	38.6	−25	45	0.36	16.7	38.6	3.4	58.0
Bernstein	1702	217	371	0.000	−251.0	−790	288	−12.9	−40.4	14.7	−48	21	0.38	14.3	36.4	60.2	3.4

WTHT: Equation including weight and height; H–B: Harris and Benedict equation; WT: equation including weight; REE: resting energy expenditure; SD standard deviation; Bias: mean difference between the measured REE and the predicted value; LLA: lower limit of agreement; ULA: upper limit of agreement; Bias %: mean bias in %; LLA %: lower limit of agreement in percentage; ULA % upper limit of agreement in percentage; CCC, concordance correlation coefficient; MAPE: mean absolute percentage error; Acc %: percentage of subjects in which the error of the predictive equation was within 10% of the measured value. Und %: percentage of subjects underestimated by the predictive equation with an error > 10% of the measured value; Over %: percentage of subjects overestimated by the predictive equation with an error > 10% of the measured value. * Equation developed for tropical populations (*n* = 86 for men and *n* = 81 for women). Equations highlighted did not include obese patients. ^&^ *p* < 0.05 better accuracy in men than women. The background colour is used to identify some of the equations.

**Table 5:** Assessment of resting energy prediction equations in overweight and obese women (*n* = 86) from the population of Gran Canaria.

	REE (kcal/d)			*t*-Test	Bias	LLA	ULA	Bias	LLA	ULA	Maximal Error		Prediction				
Equation	Mean	SD	RMSD	*p*-Value	kcal/d	kcal/d	kcal/d	%	%	%	Under %	Over %	CCC	MAPE	Acc %	Und %	Over %
Measured	1491	288													100		
Mifflin	1507	152	230	0.51	16.5	−435	468	1.1	−28.5	30.7	−37	35	0.50	12.9	52.3	17.4	30.2
Livingston	1510	113	236	0.44	19.7	−444	483	1.3	−29.0	31.6	−34	38	0.41	13.1	48.8	17.4	33.7
Henry WTHT	1523	129	240	0.21	32.5	−437	502	2.2	−28.5	32.9	−36	37	0.42	13.5	48.8	16.3	34.9
Owen	1416	75	261	0.007	−75.2	−567	417	−5.0	−36.9	26.9	−46	31	0.27	12.9	45.3	36.0	18.6
H–B 1984	1565	128	243	0.004	74.5	−382	530	5.0	−25.0	35.0	−32	39	0.43	14.1	45.3	12.8	41.9
Schofield WTHT	1568	136	256	0.005	77.2	−404	558	5.2	−26.1	36.5	−34	41	0.38	14.8	45.3	12.8	41.9
H–B 1919	1593	128	252	0.000	102.0	−353	557	6.8	−23.2	36.8	−30	41	0.41	14.9	44.2	10.5	45.3
FAO WTHT	1598	137	266	0.000	107.4	−371	586	7.2	−24.0	38.4	−32	42	0.37	15.7	44.2	10.5	45.3
Huang	1384	130	256	0.000	−107.2	−565	351	−7.2	−37.0	22.6	−48	27	0.40	12.5	44.2	40.7	15.1
WHO WTHT	1593	129	263	0.000	102.3	−374	579	6.9	−24.3	38.0	−32	42	0.37	15.5	44.2	10.5	45.3
Henry and Rees *^&^	1568	118	244	0.009	69.7	−394	533	4.7	−25.5	34.9	−38	41	0.38	13.7	43.4	13.3	43.4
Korth	1607	158	257	0.000	116.0	−337	569	7.8	−22.0	37.5	−32	40	0.45	15.2	43.0	10.5	46.5
Lazzer 2010	1585	128	249	0.000	94.3	−360	549	6.3	−23.7	36.3	−32	40	0.42	14.8	43.0	10.5	46.5
Muller	1575	136	243	0.001	83.9	−366	534	5.6	−24.0	35.3	−33	39	0.45	14.3	41.9	12.8	45.3
Kleiber	1609	168	259	0.000	118.4	−336	573	7.9	−21.8	37.7	−28	43	0.46	15.3	39.5	11.6	48.8
FAO WT	1638	134	282	0.000	147.1	−327	621	9.9	−21.1	40.8	−29	44	0.35	17.2	34.9	10.5	54.7
Bernstein	1295	90	311	0.000	−196.2	−672	280	−13.2	−43.6	17.3	−50	21	0.25	14.7	34.9	57.0	8.1
Weijs	1680	168	298	0.000	189.3	−266	644	12.7	−17.2	42.6	−32	47	0.39	18.6	32.6	7.0	60.5
De Lorenzo ^&^	1787	178	373	0.000	296.4	−150	743	19.9	−9.4	49.1	−20	51	0.31	24.2	26.7	2.3	70.9
De Luis ^&^	1870	150	444	0.000	379.6	−75	835	25.5	−4.5	55.4	−11	61	0.21	29.7	14.0	2.3	83.7

WTHT: Equation including weight and height; H–B: Harris and Benedict equation; WT: equation including weight; REE: resting energy expenditure; SD standard deviation; Bias: mean difference between the measured REE and the predicted value; LLA: lower limit of agreement; ULA: upper limit of agreement; Bias %: mean bias in %; LLA %: lower limit of agreement in percentage; ULA % upper limit of agreement in percentage; CCC, concordance correlation coefficient; MAPE: mean absolute percentage error; Acc %: percentage of subjects in which the error of the predictive equation was within 10% of the measured value. Und %: percentage of subjects underestimated by the predictive equation with an error > 10% of the measured value; Over %: percentage of subjects overestimated by the predictive equation with an error > 10% of the measured value. * Equation developed for tropical populations (*n* = 86 for men and *n* = 81 for women). Equations highlighted did not include obese patients. ^&^ *p* < 0.05 better accuracy in men than women. The background colour is used to identify some of the equations.

**Table 6:** Assessment of resting energy prediction equations using body composition data in overweight and obese adults (88 men and 86 women) from the population of GranCanaria.

	REE (kcal/d)			*t*-Test	Bias	LLA	ULA	Bias	LLA	ULA	Maximal Error		Prediction				
Equation	Mean	SD	RMSD	*p*-Value	kcal/d	kcal/d	kcal/d	%	%	%	Under %	Over %	CCC	MAPE	Acc %	Und %	Over %
REE Measured	1727	389													100		
Muller ALL	1737	265	250	0.51	12.7	−477	503	0.7	−27.0	28.5	−37	35	0.72		52.9	17.8	29.3
Muller men ^§^	1957	160	268	0.88	4.4	−524	533	0.2	−25.8	26.2	−37	35	0.47	10.7	55.7	18.2	26.1
Muller women ^§^	1512	123	229	0.40	21.1	−429	471	1.4	−28.2	31.0	−36	35	0.46	12.9	50.0	17.4	32.6
Johnstone ALL	1710	273	241	0.43	−14.5	−487	458	−0.8	−27.8	26.1	−39	37	0.74		52.9	20.7	26.4
Johnstone men	1915	205	260	0.18	−37.4	−545	470	−1.9	−27.1	23.3	−39	37	0.56	10.0	56.8	21.6	21.6
Johnstone women	1500	143	219	0.71	8.9	−423	440	0.6	−27.9	29.0	−35	35	0.53	12.3	48.8	19.8	31.4
Korth ALL	1673	295	252	0.007	−51.2	−536	434	−3.0	−30.7	24.7	−40	39	0.73		51.7	28.7	19.5
Korth men ^§^	1916	200	263	0.200	−36.1	−550	478	−1.9	−27.5	23.8	−39	39	0.54	10.3	59.1	22.7	18.2
Korth women ^§^	1424	111	240	0.009	−66.6	−521	388	−4.5	−34.2	25.2	−40	33	0.41	12.3	44.2	34.9	20.9
Lazzer 2010 ALL	1776	231	259	0.008	51.6	−446	550	3.0	−25.8	31.7	−38	42	0.67		50.6	14.9	34.5
Lazzer 2010 men ^§^	1963	161	265	0.72	10.1	−513	533	0.5	−25.5	26.5	−38	39	0.48	10.6	58.0	15.9	26.1
Lazzer 2010 women	1585	96	251	0.000	94.0	−365	554	6.3	−24.0	36.6	−28	42	0.37	15.0	43.0	14.0	43.0
Owen ALL	1440	289	380	0.000	−284.4	−780	211	−16.5	−45.1	12.1	−57	27	0.54		27.0	70.7	2.3
Owen men ^&^^¶^	1690	178	372	0.000	−262.9	−780	254	−13.5	−39.0	12.1	−51	27	0.35	14.4	30.7	65.9	3.4
Owen women ^¶^	1184	87	389	0.000	−306.4	−778	165	−20.6	−50.1	9.0	−57	14	0.18	19.4	23.3	75.6	1.2
Bernstein ALL	1330	232	467	0.000	−394.2	−885	97	−22.9	−49.2	3.5	−61	16	0.39		12.6	86.8	0.6
Bernstein men ^¶^	1513	166	511	0.000	−439.8	−954	74	−22.5	−47.4	2.3	−61	16	0.21	14.3	10.2	88.6	1.1
Bernstein women ^¶^	1143	106	416	0.000	−347.5	−799	104	−23.3	−51.3	4.7	−60	9	0.19	14.7	15.1	84.9	0.0

WTHT: Equation including weight and height; H–B: Harris and Benedict equation; WT: equation including weight; REE: resting energy expenditure; SD standard deviation; Bias: mean difference between the measured REE and the predicted value; LLA: lower limit of agreement; ULA: upper limit of agreement; Bias %: mean bias in %; LLA %: lower limit of agreement in percentage; ULA % upper limit of agreement in percentage; CCC, concordance correlation coefficient; MAPE: mean absolute percentage error; Acc %: percentage of subjects in which the error of the predictive equation was within 10% of the measured value. Und %: percentage of subjects underestimated by the predictive equation with an error > 10% of the measured value; Over %: percentage of subjects overestimated by the predictive equation with an error > 10% of the measured value. ^§^ More accurate than the respective anthropometric equation (*p* < 0.05); ^¶^ Less accurate than the respective anthropometric equation (*p* < 0.05).

## Supplementary Materials

The following are available online at https://www.mdpi.com/2077-0383/9/1/203/s1, Table S1: Predictive equations for resting energy expenditure (REE) in adults. Figure S1: Bland–Altman plots displaying the agreement between measured and predicted REE in percentage by the equations of (A) Mifflin, (B) Livingston, (C) Huang, (D) Owen, (E) Schofield WTHT, (F) Muller, (G) WHO WTHT, and (H) FAO WTHT, Figure S2: Bland–Altman plots displaying the agreement between measured and predicted REE in percentage by the equations of (A) Lazzer, (B) Kleiber, (C) Korth, (D) FAO WT, (E) De Lorenzo, (F) Weijs, (G) Bernstein, and (H) De Luis; Figure S3: Bland–Altman plots displaying the agreement between measured and predicted REE in percentage by the equations of (A) Henry and Rees (tropical populations), (B) Henry WTHT, (C) Harris-Benedict 1984, and (D) Harris-Benedict 1919, Figure S4: Bland–Altman plots displaying the agreement between measured and predicted REE by the body composition-based equations of (A) Muller, (B) Johnstone, (C) Korth, (D) Lazzer 2010, (E) Owen, and (F) Bernstein.
