# Supplementary material for: Resting Energy Expenditure and Body Composition in Overweight Men and Women Living in a Temperate Climate
Source: J Clin Med. 2020 Jan 11;9(1):203. doi: 10.3390/jcm9010203 (PMC7020055; doi:10.3390/jcm9010203)
Supplement: Supplementary file 1 [file jcm-09-00203-s001.zip › MS jcm-641682Corrected supplementary Material Tables S1 and Fig s1_s2_s3_s4/Martin-Rincon-Table S1_Revised 22-12-2019_FINAL 10-01-20.docx]

| **Table S1**. Predictive equations for resting energy expenditure (REE) in adults | | | |
| --- | --- | --- | --- |
| Author/s | Study population | Age (mean ± SD or range) | REE Equations (Kcal/day or MJ/day) |
| Bernstein et al. [1] | Obese; n: 48 M/154 F  BMI: NR | M:40±13; F:39±12  M:19 – 59; F:18-59 | M (kcal/d) = 11.02 × WT + 10.23 × HTCM - 5.8 × AGE – 1032  F (kcal/d) = 7.48 × WT - 0.42 × HTCM - 3.0 × AGE + 844  M and F (kcal/d) = 19.02 x FFM + 3.72 x FM - 1.55 x AGE + 236.7 |
| De Lorenzo et al. [2] | Healthy subjects; n: 127 M/193 F  BMI: M:19-39; F:18-40 | M:29±11; F:29±11  M:19-59; F:18-59 | M (kJ) = 46.322 × WT + 15.744 HTCM - 16.66 × AGE + 944  W (kJ) = 53.284 × WT + 20.957 HTCM - 23.859 × AGE + 487 |
| De Luis et al. [3] | Obese; n: 60 M/140 F  BMI: M:30-41; F:30-39 | M:44±15; F:47±18  M:30-60; F:29-62 | M (kcal/d) = 6.1 × WT + 1023.7 HTM - 9.5 × AGE + 58.6  F (kcal/d) = 9.8 × WT + 61.6 × HTM – 8.2 × AGE + 1272.5 |
| Harris and Benedict 1919 [4] | Most of them young and healthy non-obese;  n: 136 M/103 F | M:27±9; F:31±14  M: 16-63; F:15-74 | M (kcal/d) = 13.7516 × WT + 5.0033 × HTCM – 6.755 × AGE + 66.755  F (kcal/d) = 9.5634 × WT + 1.8496 × HTM – 4.6756 × AGE + 655.0955 |
| Harris and Benedict 1984 [5] | Most young and healthy non-obese; n: 168 M/169 F | M:30±14; F:40±22 | M (kcal/d) = 13.397 × WT + 4.799 × HTCM – 5.677 × AGE + 88.362  F (kcal/d) = 9.247 × WT + 3.098 × HTCM – 4.33 × AGE + 447.593 |
| FAO/WHO/UNU [6] | n: 575 M/734 F | All: 30–82 | Equations based on weight  M 18–30 (kcal/d) = (15.3 × WT) + 679  F 18–30 (kcal/d) = 14.7 × WT) + 496  M 30–60 (kcal/d) = (11.6 × WT) + 879  F 30–60 (kcal/d) = (8.7 × WT) + 829  M 60 (kcal/d) = (13.5 × WT) + 487  F 60+ (kcal/d) = (10.5 × WT) + 596  *Equations based on weight and height*  M 18–30 (kcal/d) = (15.4 × WT) – (27 × HTM) + 717  F 18–30 (kcal/d) = (13.3 × WT) + (334 × HTM) + 35  M 30–60 (kcal/d) = (11.3 × WT) + (16 × HTM) + 901  F 30–60 (kcal/d) = (8.7 × WT) - (25 × HTM) + 865  M 60+ (kcal/d) = (8.8 × WT) + (1128 × HTM) – 1071  F 60+ (kcal/d) = (9.2 × WT) + (637 × HTM) – 302 |
| Henry [7] | 18–30 y: 2816 M/1655 F  BMI: 20.9±2.8 M // 20.7±3.2 F  30–60 y: 1006 M/1023 F  BMI: 22.8±3.2 M // 23.3±4.5 F  60+ y: 533 M/324 F | 18- +60 | M 18–30 y (kcal/d) = 14.4 × WT + 313 × HTM + 113  F 18–30 y (kcal/d) = 10.4 × WT + 615 × HTM – 282  M 30–60 y (kcal/d) = 11.4 × WT + 541 × HTM – 137  F 30–60 y (kcal/d) = 8.18 × WT + 502 × HTM – 11.6  M 60+ y (kcal/d) = 11.4 × WT + 541 × HTM – 256  F 60+ y (kcal/d) = 8.52 × WT + 421 × HTM + 10.7 |
| Henry and Rees [8] | Mostly Asians living in tropical weather  18–30 y: 1174 M/350 F  BMI: NR  30–60 y: 274 M/98 F  BMI: NR | 18-60 | M 18–30 y (MJ/d) = 0.057 × WT – 0.429 × HTM + 3.412  F 18–30 y(MJ/d) = 0.042 × WT + 1.546 × HTM + 0.433  M 30–60 y (MJ/d) = 0.046 × WT – 0.081 × HTM + 3.277  F 30–60 y (MJ/d) = 0.047 × WT + 0.145 × HTM + 2.256 |
| Huang et al. [9] | 142 Diabetics (61 M/81 F)  BMI: 48.0±7.9 M // 47.4±8.8 F  896 Nondiabetic (218 M/678 F)  BMI: 42.5±7.4 M // 46.0±8.2 F | Diabetics age  M: 52±12; F:52±12  Nondiabetics age  M: 44±13; F:44±12 | ALL (kcal/d) = 9.996 × WT + 4.231 × HTCM - 2.337 × AGE + 257.293 × SEX + 71.767 |
| Kleibler [10] | 136 M; 68 BW (mean)=56.3; 68 M BW (mean) = 71.9  103 W Studied in the Carnegie Nutrition Laboratory | NR | M (kcal/d) = 71.2 × WT^0.75^ × (1 + 0.004 × [30 - AGE] + 0.01 × [HTCM/WT^0.33^ – 43.4])  F (kcal/d) = 65.8 × WT^0.75^ × (1 + 0.004 × [30 - AGE] + 0.018 × [HTCM/WT^0.33^ - 42.1]) |
| Korth et al. [11] | n: 50 M/54 F  BMI: 21-36 M // 18-41 F | M: 39±15; F:35±15  M: 21-68; F: 21-66 | ALL (kJ/d) = 41.5 × WT - 35.0 × HTCM - 19.1 × AGE +  1107.4 × SEX - 1731.2  (SEX: male = 1; female = 0)  ALL (kJ/d) = 105.1 x FFM + 1422 * |
| Livingston and Kohlstadt [12] | Normal weight and obese  n: 299 M/356 F; BMI: NR | M: 39±13; F:36±15  M: 18-95; F: 18-77 | M (Kcal/d) = 293 × WT ^0.4330^ - 5.92 × AGE  F (Kcal/d) = 248 × WT ^0.4356^ - 5.09 × AGE |
| Mifflin et al. [13] | Normal weight (n: 264) and obese (n:234) n: 251 M/247 F;  BMI: 19-42 M // 17-42 F | M: 44±14; F:45±14  M: 19-76; F: 20-76 | M (Kcal/d) = 9.99 × WT + 6.25 × HTCM - 4.92 × AGE + 5  F (Kcal/d) = 9.99 × WT + 6.25 × HTCM - 4.92 × AGE - 161 |
| Muller et al. [14] | Data from seven different research centres in Germany  BMI 25-30, n: 101 M/ 165 F  BMI ≥ 30, n: 99 M/ 179 F | BMI 25-30: 54±16.0  BMI ≥ 30: 48±14 | All (MJ/d) = 0.047 × WT - 0.01452 × AGE + 1.009 × SEX + 3.21  BMI 25–30 (MJ/d) = 0.04507 × WT + 1.006 × SEX - 0.01553 × AGE + 3.407  BMI 25–30 (MJ/d) = 0.03776 x FFM + 0.03013 x FM + 0.93 x sex - 0.01196 x AGE + 3.928  BMI ≥ 30 (MJ/d) = 0.05 × WT + 1.103 × SEX - 0.01586 × AGE + 2.924  BMI ≥ 30 (MJ/d) = 0.05685 x FFM + 0.04022 x FM + 0.808 x sex - 0.01402 AGE + 2.818  (SEX male = 1; female = 0) |
| Schofield [15] | Collection of different authors and  papers  M 18–30 y: 2879 M/829 F  M 30–60 y: 646 M/372 F  M 60+ y: 50 M/38 F | 18- +60 | M 18–30 y (MJ): 0.063 × WT – 0.042 × HTM + 2.953  F 18–30 y(MJ): 0.057 × WT + 1.184 × HTM + 0.411  M 30–60 y (MJ): 0.048 × WT - 0.011 × HTM + 3.67  F 30–60 y (MJ): 0.034 × WT + 0.006 × HTM + 3.53  M 60+ y (MJ): 0.038 × WT + 4.068 × HTM - 3.491  F 60+ y (MJ): 0.033 × WT + 1.917 × HTM + 0.074 |
| Weijs and Vansant [16] | 25 men; BMI 25–30 [28.1 (1.4)]  29 men; BMI 30–40 [33.3 (2.4)]  80 women; BMI 25–30 [27.9 (1.4)]  74 women; BMI 30–40 [34.0 (2.6)] | 43±13  41±12  40±12  41±12 | REE (kcal/d) = 14.038 × WT + 4.498 × HTCM + 137.566 × SEX - 0.977 × AGE - 221.631  (SEX: male = 1; female = 0) |
| WHO [6,17] (Weight and Height) | n = 8716 (normal weight, overweight and obese) | 18- +60 | M 18–30 y (kJ/d) = 64.4 × WT – 113 × HTM + 3000  F 18–30 y (kJ/d) = 55.6 × WT + 1397.4 × HTM + 146  M 30–60 y (kJ/d) = 47.2 × WT + 66.9 × HTM + 3769  F 30–60 y (kJ/d) = 36.4 × WT - 104.6 × HTM + 3619  M 60+ y (kJ/d) = 36.8 × WT + 4719.5× HTM - 4481  F 60+ y (kJ/d) = 38.5 × WT + 2665 × HTM -1264 |
| Lazzer et al. 2007 [18,19] | n = 164 severely obese men (BMI: 35- >50)  n = 182 severely obese women (BMI: 40 - >50) | 20-65  19-60 | REE (MJ/d) = 0.048 x WT + 4.655 x HTM - 0.020 x AGE - 3.605  REE (MJ/d) = 0.081 x FFM + 0.049 x FM – 0.019 x AGE - 2.194  REE (MJ/d) = 0.042 x WT + 3.619 x HTM - 2.678  REE (MJ/d) = 0.067 x FFM + 0.046 x FM + 1.568 |
| Lazzer et al. 2010 [20] |  |  |  |
| Owen et al. [21,22] | n = 60 men (BMI 20-59)  n = 44 women (BMI 18-50) | 18-82  18-65 | REE (kcal/d) = 879 + 10.2 x WT  REE (kcal/d) = 290 + 22.3 x FFM  REE (kcal/d) = 795 + 7.18 x WT  REE (kcal/d) = 334 + 19.7 x FFM |
| Johnstone et al. [23] | n = 43 men (n = 16; BMI 23.2 ± 1.2; n =20 BMI 26.8 ± 1.4; n= 7; BMI 38.1 ± 0.8)  n = 107 women (n = 11; BMI 18.9 ± 1.0; n =42 BMI 22.4 ± 1.4; n= 32; BMI 26.6 ± 1.4; n = 22 BMI 36.3 ± 16.5) | 47±10  42±11 | REE (kJ/d) = 90.2 x FFM + 31.6 x FM - 12.2 × AGE + 1613 |
| BMI: body mass index (kg.m^-2^); NR: not reported; M: males; F: females; WT: weight in kg; HTCM: height in cm; HTM: height in m. The equations in blue colour did not include obese subjects. FFM: fat-free mass; FM: fat mass. The equations predicting in Joules were converted to cal by applying the conversion factor 1 cal = 4.184 J. * Equation specific for DXA-measured FFM. | | | |

**References**

1. Bernstein, R.S.; Thornton, J.C.; Yang, M.U.; Wang, J.; Redmond, A.M.; Pierson, R.N., Jr.; Pi-Sunyer, F.X.; Van Itallie, T.B. Prediction of the resting metabolic rate in obese patients. *Am. J. Clin. Nutr.* **1983**, *37*, 595-602.

2. De Lorenzo, A.; Tagliabue, A.; Andreoli, A.; Testolin, G.; Comelli, M.; Deurenberg, P. Measured and predicted resting metabolic rate in italian males and females, aged 18-59 y. *Eur. J. Clin. Nutr.* **2001**, *55*, 208-214.

3. de Luis, D.A.; Aller, R.; Izaola, O.; Romero, E. Prediction equation of resting energy expenditure in an adult spanish population of obese adult population. *Ann. Nutr. Metab.* **2006**, *50*, 193-196.

4. Harris, J.A.; Benedict, F.G. A biometric study of human basal metabolism. *Proc. Natl. Acad. Sci. U. S. A.* **1918**, *4*, 370-373.

5. Roza, A.M.; Shizgal, H.M. The harris benedict equation reevaluated: Resting energy requirements and the body cell mass. *Am. J. Clin. Nutr.* **1984**, *40*, 168-182.

6. FAO/WHO/UNU. Human energy requirements: Report of a joint fao/who/unu expert consultation. Fao food nutr tech rep ser [internet]. 2001;0:96. Available from: <Ftp://ftp.Fao.Org/docrep/fao/007/y5686e/y5686e00.Pdf>. *World Health Organ. Tech. Rep. Ser.* **1985**, *724*, 1-206.

7. Henry, C.J. Basal metabolic rate studies in humans: Measurement and development of new equations. *Public Health Nutr.* **2005**, *8*, 1133-1152.

8. Henry, C.J.; Rees, D.G. New predictive equations for the estimation of basal metabolic rate in tropical peoples. *Eur. J. Clin. Nutr.* **1991**, *45*, 177-185.

9. Huang, K.C.; Kormas, N.; Steinbeck, K.; Loughnan, G.; Caterson, I.D. Resting metabolic rate in severely obese diabetic and nondiabetic subjects. *Obes. Res.* **2004**, *12*, 840-845.

10. Keibler, M. Body size and metabolism. *Hilgardia* **1932**, *11*, 315-353.

11. Korth, O.; Bosy-Westphal, A.; Zschoche, P.; Gluer, C.C.; Heller, M.; Muller, M.J. Influence of methods used in body composition analysis on the prediction of resting energy expenditure. *Eur. J. Clin. Nutr.* **2007**, *61*, 582-589.

12. Livingston, E.H.; Kohlstadt, I. Simplified resting metabolic rate-predicting formulas for normal-sized and obese individuals. *Obes. Res.* **2005**, *13*, 1255-1262.

13. Mifflin, M.D.; St Jeor, S.T.; Hill, L.A.; Scott, B.J.; Daugherty, S.A.; Koh, Y.O. A new predictive equation for resting energy expenditure in healthy individuals. *Am. J. Clin. Nutr.* **1990**, *51*, 241-247.

14. Muller, M.J.; Bosy-Westphal, A.; Klaus, S.; Kreymann, G.; Luhrmann, P.M.; Neuhauser-Berthold, M.; Noack, R.; Pirke, K.M.; Platte, P.; Selberg, O.*, et al.* World health organization equations have shortcomings for predicting resting energy expenditure in persons from a modern, affluent population: Generation of a new reference standard from a retrospective analysis of a german database of resting energy expenditure. *Am. J. Clin. Nutr.* **2004**, *80*, 1379-1390.

15. Schofield, W.N. Predicting basal metabolic rate, new standards and review of previous work. *Hum. Nutr. Clin. Nutr.* **1985**, *39 Suppl 1*, 5-41.

16. Weijs, P.J.; Vansant, G.A. Validity of predictive equations for resting energy expenditure in belgian normal weight to morbid obese women. *Clin. Nutr.* **2010**, *29*, 347-351.

17. Madden, A.M.; Mulrooney, H.M.; Shah, S. Estimation of energy expenditure using prediction equations in overweight and obese adults: A systematic review. *J. Hum. Nutr. Diet.* **2016**, *29*, 458-476.

18. Lazzer, S.; Agosti, F.; Resnik, M.; Marazzi, N.; Mornati, D.; Sartorio, A. Prediction of resting energy expenditure in severely obese italian males. *J. Endocrinol. Invest.* **2007**, *30*, 754-761.

19. Lazzer, S.; Agosti, F.; Silvestri, P.; Derumeaux-Burel, H.; Sartorio, A. Prediction of resting energy expenditure in severely obese italian women. *J. Endocrinol. Invest.* **2007**, *30*, 20-27.

20. Lazzer, S.; Bedogni, G.; Lafortuna, C.L.; Marazzi, N.; Busti, C.; Galli, R.; De Col, A.; Agosti, F.; Sartorio, A. Relationship between basal metabolic rate, gender, age, and body composition in 8,780 white obese subjects. *Obesity* **2010**, *18*, 71-78.

21. Owen, O.E.; Holup, J.L.; D'Alessio, D.A.; Craig, E.S.; Polansky, M.; Smalley, K.J.; Kavle, E.C.; Bushman, M.C.; Owen, L.R.; Mozzoli, M.A.*, et al.* A reappraisal of the caloric requirements of men. *Am. J. Clin. Nutr.* **1987**, *46*, 875-885.

22. Owen, O.E.; Kavle, E.; Owen, R.S.; Polansky, M.; Caprio, S.; Mozzoli, M.A.; Kendrick, Z.V.; Bushman, M.C.; Boden, G. A reappraisal of caloric requirements in healthy women. *Am. J. Clin. Nutr.* **1986**, *44*, 1-19.

23. Johnstone, A.M.; Rance, K.A.; Murison, S.D.; Duncan, J.S.; Speakman, J.R. Additional anthropometric measures may improve the predictability of basal metabolic rate in adult subjects. *Eur. J. Clin. Nutr.* **2006**, *60*, 1437-1444.
