# Supplementary material for: Resting Energy Expenditure and Body Composition in Overweight Men and Women Living in a Temperate Climate
Source: J Clin Med. 2020 Jan 11;9(1):203. doi: 10.3390/jcm9010203 (PMC7020055; doi:10.3390/jcm9010203)
Supplement: Supplementary file 1 [file jcm-09-00203-s001.zip › MS jcm-641682Corrected supplementary Material Tables S1 and Fig s1_s2_s3_s4/Supplementary Figures S1, S2, S3 and S4_Corrected 10-01-20.docx]

.
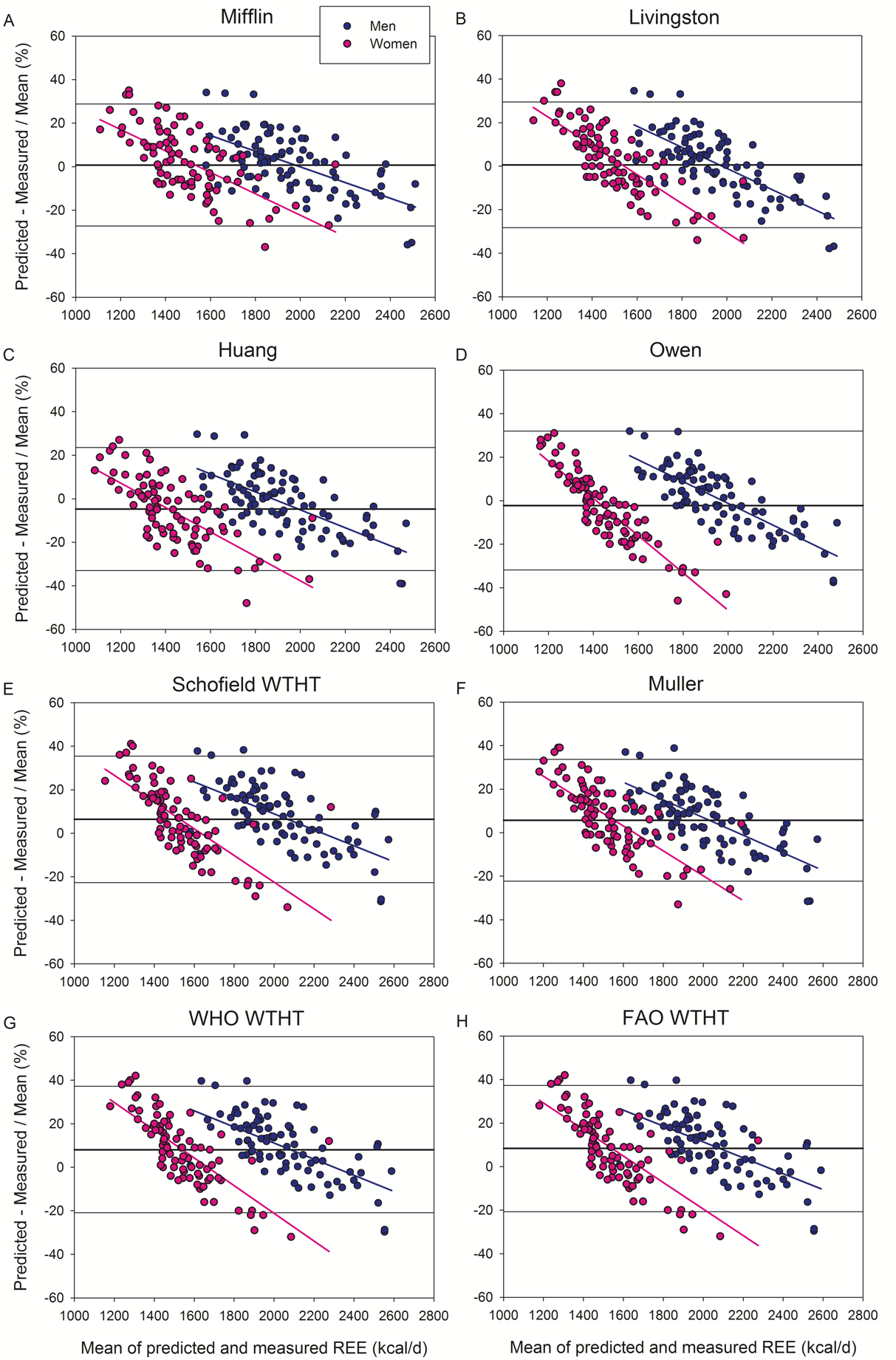


**Figure S1.** Bland–Altman plots displaying the agreement between measured and predicted REE in percentage by the equations of (A) Mifflin, (B) Livingston, (C) Huang, (D) Owen, (E) Schofield WTHT, (F) Muller, (G) WHO WTHT, and (H) FAO WTHT. All these equations include obese subjects in their populations. The thick continuous line indicates the mean value of the differences between predicted and measured REE (bias). The thin lines delimit the 95% confidence interval. All the regression lines were statistically significant at P < 0.001, indicating a systematic bias. Note that the “X” axes have different scales.


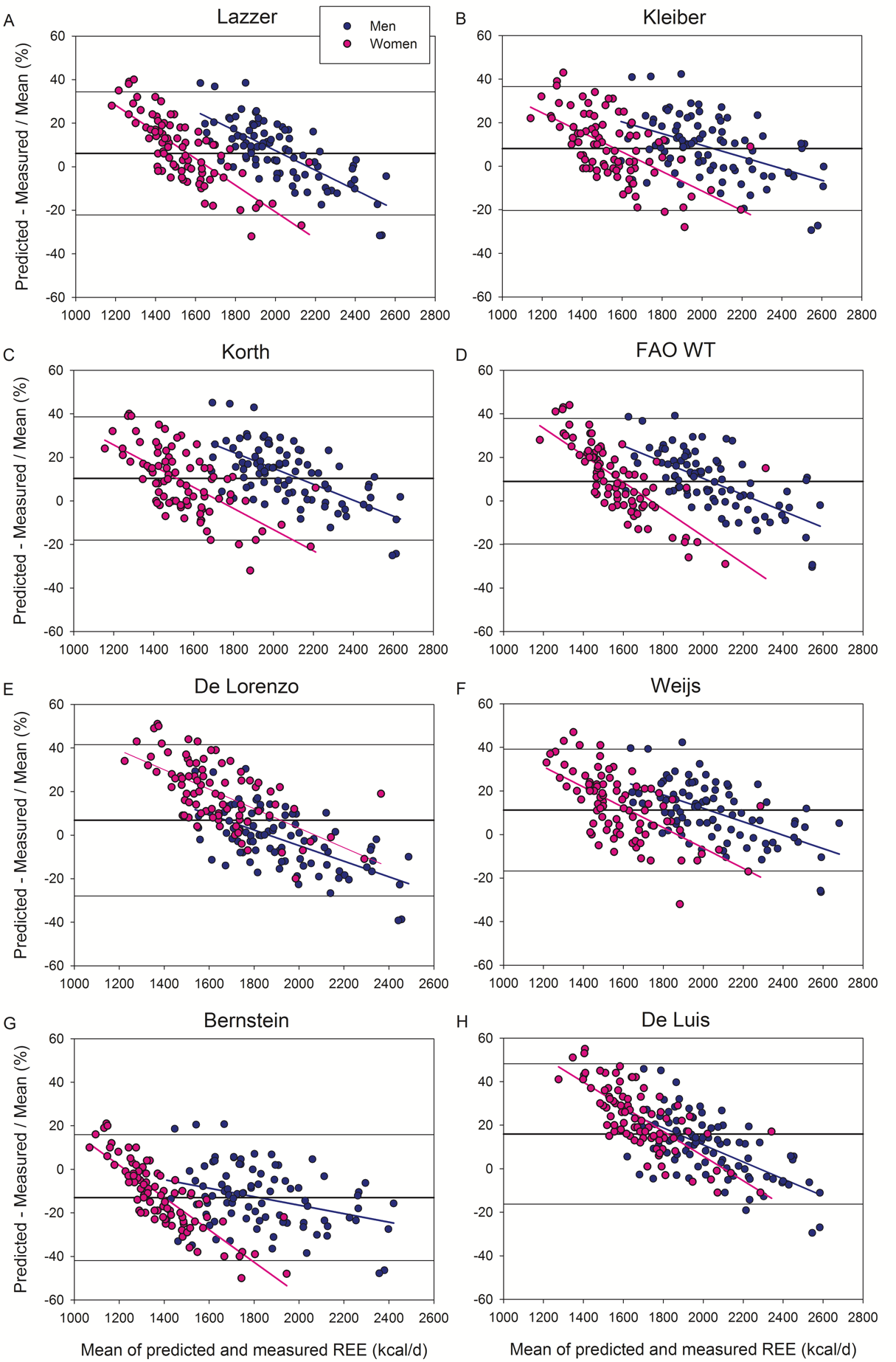


**Figure S2.** Bland–Altman plots displaying the agreement between measured and predicted REE in percentage by the equations of (A) Lazzer, (B) Kleiber, (C) Korth, (D) FAO WT, (E) De Lorenzo, (F) Weijs, (G) Bernstein, and (H) De Luis. All these equations include obese subjects in their populations. The thick continuous line indicates the mean value of the differences between predicted and measured REE (bias). The thin lines delimit the 95% confidence interval. All the regression lines were statistically significant at P < 0.001, indicating a systematic bias. Note that the “X” axes have different scales. De Luis equation was obtained using a portable, hand-held device (MedGem) less accurate than metabolic carts.

.


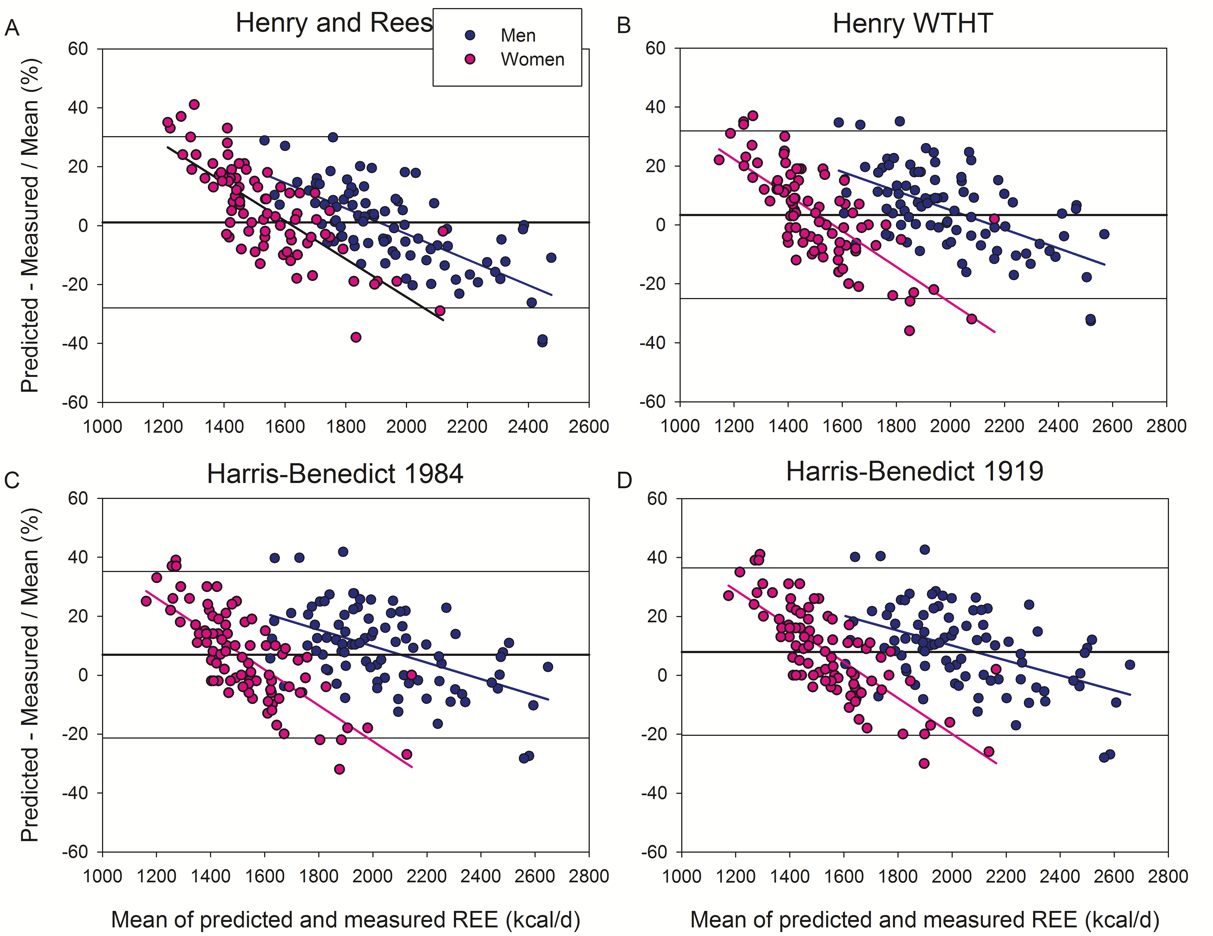


**Figure S3.** Bland–Altman plots displaying the agreement between measured and predicted REE in percentage by the equations of (A) Henry and Rees (tropical populations), (B) Henry WTHT, (C) Harris-Benedict 1984, and (D) Harris-Benedict 1919. All these equations did not include obese subjects in their populations. The thick continuous line indicates the mean value of the differences between predicted and measured REE (bias). The thin lines delimit the 95% confidence interval. All the regression lines were statistically significant at P < 0.001, indicating a systematic bias. Note that the “X” axes have different scales.


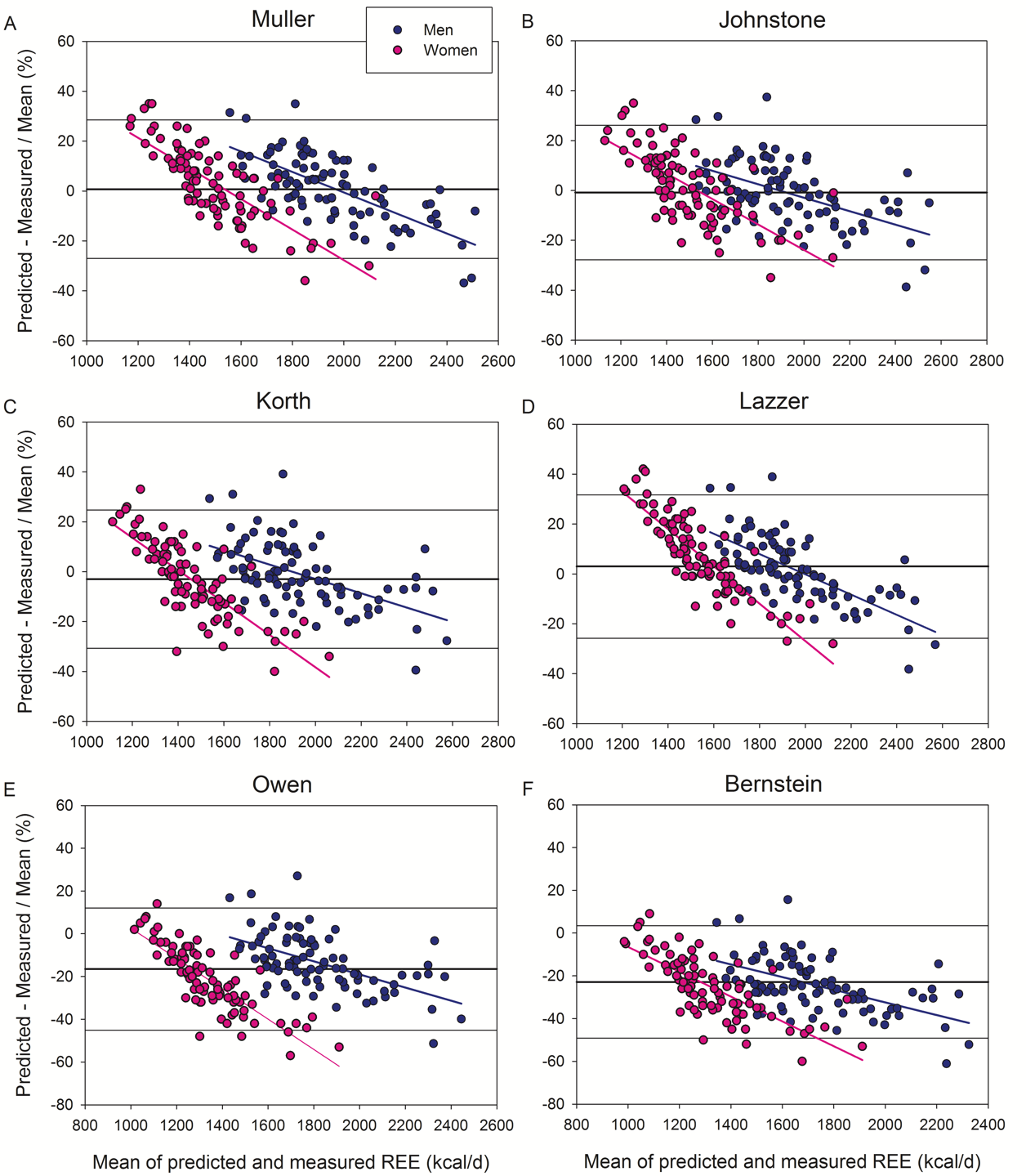


**Figure S4.** Bland–Altman plots displaying the agreement between measured and predicted REE by the body composition-based equations of (A) Muller, (B) Johnstone, (C) Korth, (D) Lazzer 2010, (E) Owen, and (F) Bernstein. All these equations include obese subjects in their populations. The thick continuous line indicates the mean value of the differences between predicted and measured REE (bias). The thin lines delimit the 95% confidence interval. All the regression lines were statistically significant at P < 0.001, indicating a systematic bias. Note that the “X” axes have different scales.
